# Integrating species-centric and geomorphic-centric views of interior least tern and piping plover habitat selection

**DOI:** 10.1016/j.heliyon.2018.e00648

**Published:** 2018-06-05

**Authors:** Jason M. Farnsworth, David M. Baasch, Patrick D. Farrell

**Affiliations:** Platte River Recovery Implementation Program, 4111 4^th^ Avenue, Suite 6, Kearney, Nebraska 68845, United States

**Keywords:** Earth sciences, Ecology

## Abstract

The Federally endangered interior least tern (*Sterna antillarum athalassos*) and threatened piping plover (*Charadrius melodus*) nest on emergent sandbars in several braided rivers in the USA. Previous habitat selection and geomorphic investigations identified a relationship between channel width and nesting incidence. Species-centric analyses indicate selection for the widest available channels whereas geomorphic-centric analyses indicate the probability of species occurrence was higher in narrow channels that better supported suitable sandbar habitat. Given the disparate conclusions from each of these perspectives, we examined species use in relation to channel-width metrics across segments of the Platte, Niobrara, and Loup Rivers from both perspectives. We found the probability of nesting incidence increased with increased maximum unvegetated channel width in all river segments. However, maximum unvegetated width decreased with increased total channel width once total width exceeded 300 m in the central Platte River and 500 m in all other river segments as did the probability that the channel was free of permanently-vegetated islands. Channels within the Lower Platte, Loup and Niobrara River systems with total widths of 500–800 m appear to be both wide enough to have a high probability of nesting incidence and narrow enough to be free of vegetated islands. Actions that affect channels with total, bank-to-bank widths of <500 m and >800 m would likely have a small influence on species use while actions that change the width characteristics of 500–800 m channels could have a strong negative or positive influence on species use. Integrating species- and geomorphic-centric views into a single analysis provided a fuller picture of the relationship between species use and channel-width metrics.

## 1 Introduction

**Fig. 1:**
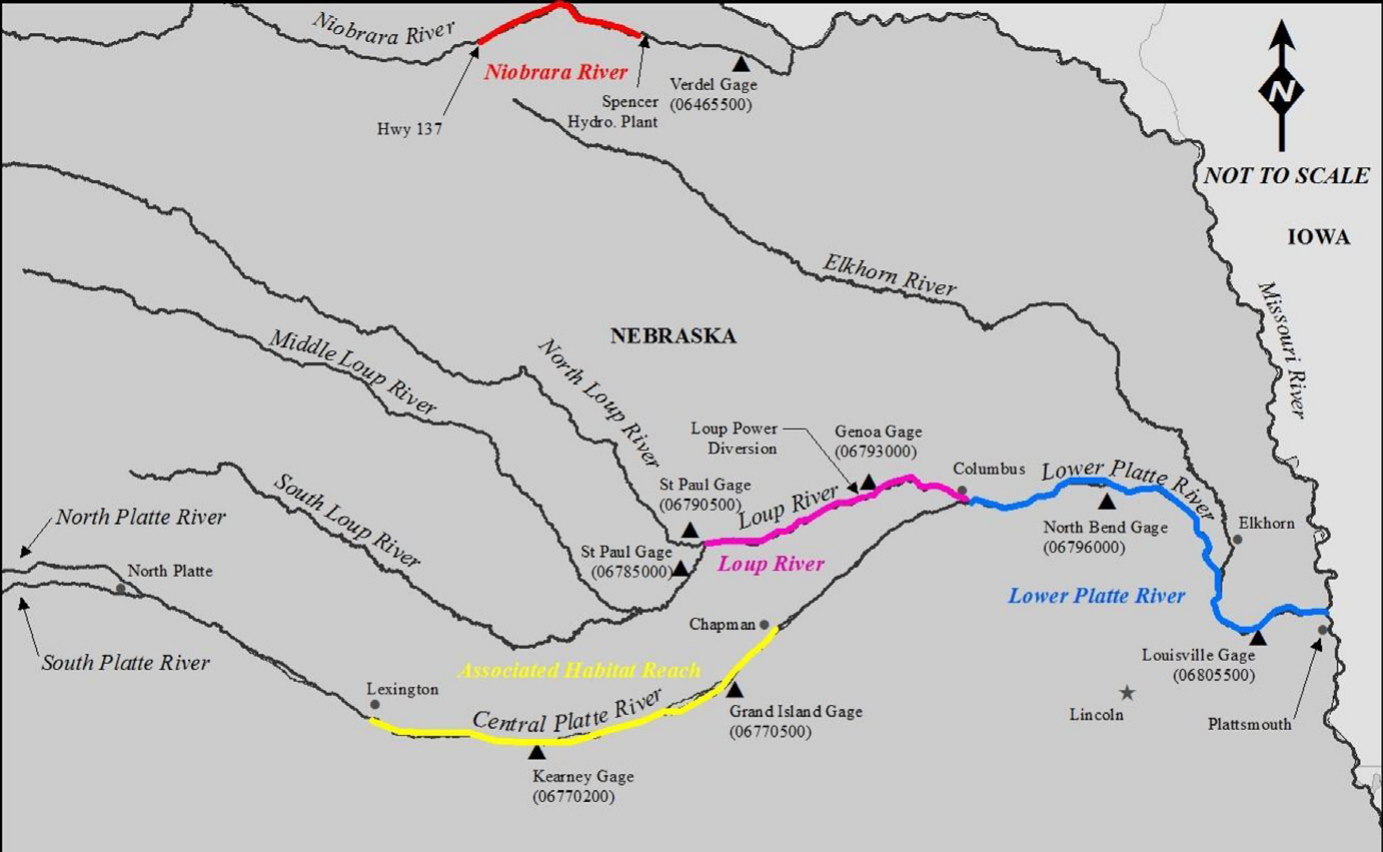
Study location map showing analysis segments on the Niobrara, Loup, lower Platte River and the Associate Habitat Reach of the central Platte River.

The Federally endangered interior least tern (*Sternula antillarum athalassos*; hereafter, least tern) and threatened piping plover (*Charadrius melodus*) nest on emergent sandbar habitat present in several braided river systems in the USA (Fig. 1; USFWS, 1988, 1990; National Research Council, 2005). Resource managers and conservationists have long been concerned about the impacts of basin water development on the habitats used by these species (Williams, 1978; Faanes, 1983; Sidle et al., 1989; Johnson, 1994; Kirsch, 1996; Murphy et al., 2004; USFWS, 2006; Joeckel and Henebry, 2008). Substantial resources have recently been spent by the Platte River Recovery Implementation Program (PRRIP) examining sandbar dynamics and evaluating sandbar height in relation to peak flow stage and the probability of sandbar inundation during the species' nesting seasons (Farnsworth et al., 2017). The PRRIP's intense focus on sandbar height led to stakeholder concerns that too much emphasis was being placed on sandbar height when several analyses identified channel width as an important variable for determining least tern and piping plover nest initiation (Ziewitz et al., 1992; Elliott, 2011; Jorgensen et al., 2012).

**Fig. 2:**
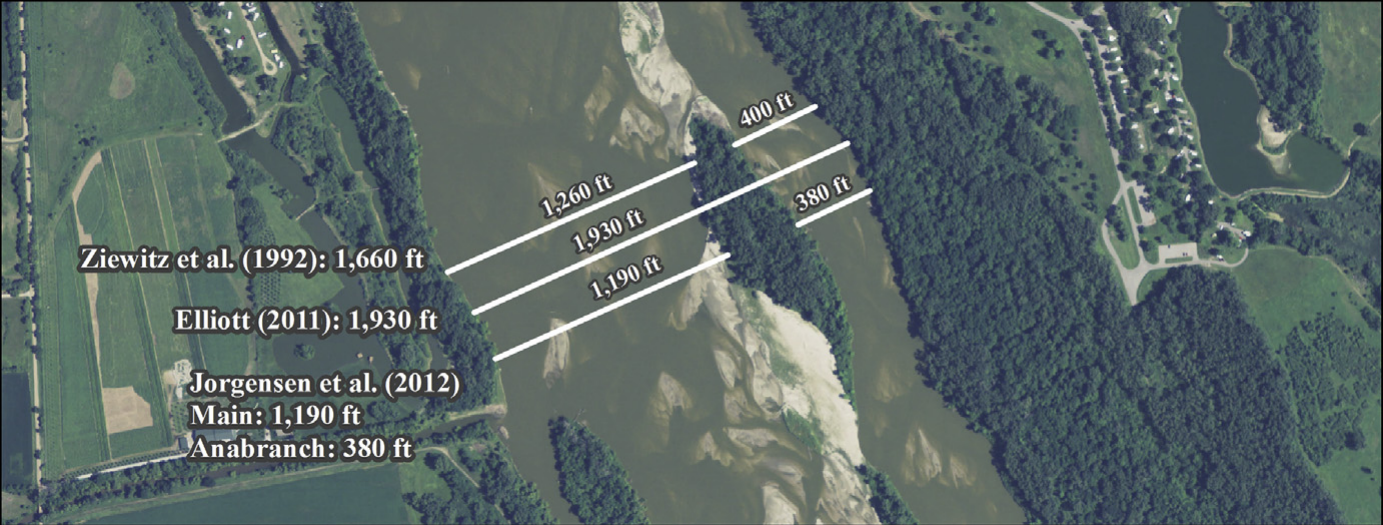
Examples of different width definitions of in-channel width measurements. The channel width in the example ranged from 116 m (Jorgensen et al., 2012 definition) to 588 m (Elliott, 2011 definition). 2009 Farm Service Agency (FSA) National Aerial Imagery Program (NAIP) aerial imagery was used to create the figure.

Ziewitz et al. (1992) performed a habitat selection analysis for 40 nest sites and defined channel width as the total width of the channel free of permanent vegetation (Fig. 2). They found average channel width, as defined, at central Platte River (CPR) and lower Platte River (LPR) nest sites was significantly greater than the mean width at a systematic sample of available sites (CPR: 295 m vs. 201 m; LPR: 519 m vs. 430 m). Jorgensen et al. (2012) also investigated the relationship between channel width and nesting incidence using a transect-based, logistic regression approach. They defined channel width as the distance between left and right channel banks, but treated channel segments split by vegetated islands as separate channels (Fig. 2). They also found a strong relationship between nesting incidence and channel width where the probability of nesting was low (<0.03) when channel widths were ≤327 m and increased sharply as channel width increased with 610 m wide channels having the highest probability of nesting (>0.80).

Elliott (2011) performed a geomorphic classification of the lower segment of the Platte River below the Loup River confluence and evaluated species nest occurrence in relation to geomorphic groupings. Elliott (2011) defined total channel width as the distance between left and right channel banks including permanently vegetated islands (Fig. 2). She found least tern and piping plover nest sites occurred disproportionally in narrower reaches of the LPR without permanently vegetated islands in 2006–2008. This result led to the conclusion that narrow channels provided ample sediment transport capacity for sandbar maintenance and likely furnished the most opportunity for providing least tern and piping plover nesting habitat in the LPR (Elliott, 2011).

Each of these investigations had unique objectives and employed different definitions of channel width, which in turn influenced the authors' interpretations of the relationships between species' use and channel width. For the purposes of this investigation, the two least tern and piping plover habitat-selection analyses conducted by Ziewitz et al. (1992) and Jorgensen et al. (2012) were considered to be species-centric as they focused on identifying physical characteristics at use locations and not on an exploration of the underlying physical process relationships driving the formation and maintenance of physical habitat used by the species. In contrast, studies such as Elliott's (2011) geomorphic classification that focused on the physical process relationships driving the formation and maintenance of physical habitat used by the species were considered to be geomorphic-centric.

The species-centric Ziewitz et al. (1992) and Jorgensen et al. (2012) investigations concluded these birds used the widest available channels, indicating actions that reduce channel width would reduce habitat suitability. In contrast, the geomorphic-centric analysis conducted by Elliott (2011) concluded narrower channels with less potential for occurrence of permanently vegetated islands supported the conditions needed for species nesting. Taken independently, these analyses have the potential to lead regulators and decision makers to very different interpretations of the channel configurations supporting least tern and piping plover nesting and the activities that may negatively influence the availability or suitability of those channels.

In this investigation, we evaluated species- and geomorphic-centric views of channel width to provide decision-makers a tool that explicitly addresses both viewpoints. Our objectives were to evaluate least tern and piping plover nest-site selection in relation to both total channel width (geomorphic-centric metric) and maximum unvegetated channel width (species-centric habitat metric) and to evaluate the relationship between these width metrics across segments of the Platte, Niobrara, and Loup Rivers that were used by these species. The analyses were conducted with the clear understanding the species also select nest sites based on non-width related metrics including the presence of emergent sandbar habitat. As such, our results are contingent on the understanding that width may be a necessary, but insufficient condition for least tern and piping plover nest initiation.

## 2 Methods

### 2.1 Study areas

The four study areas included river segments from three regional river systems in Nebraska that have been utilized by least tern and piping plover for nesting (Fig. 1). The 166 km lower Platte River (LPR) study area extended from the confluence of the Loup River downstream to the Missouri River confluence. The 64 km Niobrara River study area extended from State Highway 137 downstream to the Spencer Hydropower plant. The 116 km Loup River study area extended from the confluence of the Middle and North Loup Rivers downstream to the confluence with the Platte River at Columbus. The AHR of the central Platte River (CPR) study area included a 145 km reach extending from Lexington, NE downstream to Chapman, NE, USA. The CPR study area was excluded from the analysis of the relationship between channel width and nest incidence because species use sites were confined to mechanically-created habitats in three short river segments. However, all four study areas were included in our efforts to establish a relationship between total channel width and maximum unvegetated channel width.

### 2.2 Nest data

Least tern and piping plover nest and colony location data were obtained from several sources. Niobrara study area nesting colony locations for the period of 2005–2013 were provided by Jim Jenniges, biologist with Nebraska Public Power District (personal communication, 2014). LPR study colony locations for the period of 2008–2013 were provided by Tern and Plover Conservation Partnership and the Nongame Bird Program of the Nebraska Game and Parks Commission and were reported in their joint annual reports (Brown and Jorgensen, 2008, 2009, 2010; Brown et al., 2011, 2012, 2013). Loup River study area nest locations were obtained for the period of 2010–2012 from USFWS reports (Lackey and Runge, 2010; Lackey, 2011, 2012). Central Platte River (CPR) study area nest locations for the period of 2007–2013 were collected by the authors through implementation of the Platte River Recovery Implementation Program's (PRRIP) system scale tern and plover monitoring protocol (PRRIP, 2011a).

In all study areas, location data were collected via handheld global positioning system (GPS) units. The positional accuracy of handheld GPS units was not reported, but modern commercial-grade GPS units typically have a horizontal accuracy of ≤3 meters. The accuracy of commercial-grade GPS units was determined to be acceptable for this analysis given the objective of investigating channel width metrics at a scale of hundreds of meters. LPR nest locations were provided to the authors as river-mile locations with an accuracy of 0.1 mile (161 m). GPS locations were provided for the Niobrara, Loup and CPR study segments. Loup and CPR nest data were generalized to colony locations using a geographic information system (GIS) to identify a point at the approximate center of nest locations on individual sandbars. LPR nest location data included a colony identifier for each nest location. LPR colony locations were calculated as the average 0.1 river-mile value for all nests with the same colony identifier.

Least terns and piping plovers nested together at approximately 44% of Loup River sites, 52% of LPR sites, and 71% of Niobrara River sites included in our analysis. Almost all sites with single species nesting were occupied by least tern colonies and piping plovers were seldom observed nesting at sites without least terns. Given these species' affinity for nesting together, this analysis defines a use site as a colony location used by either or both species.

### 2.3 Aerial imagery

Channel-width measures for the LPR, Niobrara and Loup River study areas were estimated from aerial imagery collected by the Farm Service Agency (FSA) National Aerial Imagery Program (NAIP). Imagery was gathered during the months of June and July and provided data coverage for all study areas. NAIP imagery was not collected annually, however, which resulted in the occasional need to use one imagery dataset for two analysis years. We deemed this acceptable given there is little change in the area or distribution of permanently vegetated islands between years (Jorgensen et al., 2012). Channel-width metrics within the CPR study area were measured using aerial imagery collected under the PRRIP's remote sensing data collection protocol (PRRIP, 2011b).

### 2.4 Channel-width measurements

**Fig. 3:**
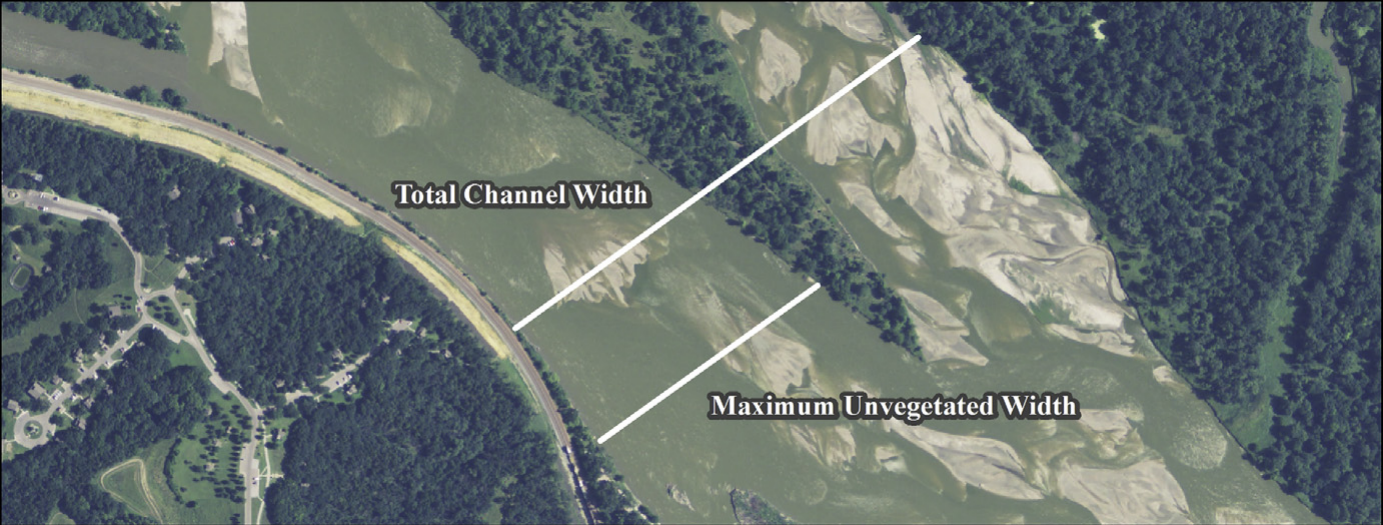
Example of how total channel width and maximum unvegetated channel width metrics were measured. 2009 Farm Service Agency (FSA) National Aerial Imagery Program (NAIP) aerial imagery was used to create the figure.

Channel widths were measured using ArcMAP geographic information system (GIS) software (Environmental Systems Research Institute, Redlands, California). Measurements at systematic locations were made perpendicular to the direction of flow at approximately 305 m intervals for each year (hereafter referred to as “available sites”). Channel-width measurements were also developed at each species use site in the Lower Platte, Niobrara and Loup River study areas. Two width measurements were recorded at each use and available site including total channel width and maximum unvegetated channel width (Fig. 3). Total channel width was defined as the total distance from apparent left bank to apparent right bank and included permanently vegetated islands which was consistent with the total channel-width definition used by Elliott (2011). Maximum unvegetated channel width at available sites was calculated as the longest contiguous unvegetated channel width from apparent left bank to apparent right bank. This was similar to the Jorgensen et al. (2012) definition of active channel width, except that the shorter unvegetated channel-width segments along individual transects were not included as additional independent transects in our analyses. Maximum unvegetated channel width at use sites was calculated as the contiguous unvegetated channel width at the nesting colony location.

Approximately 40% of the central Platte River study area has river channels that are split by up to 2-km wide and 10-km long permanent islands resulting in one main and one or more side channels. Similar conditions do not occur in our other three study areas (Murphy et al., 2004). As such, in reaches where the channel was split by these large permanent islands, channel-width measurements at available sites within the CPR study area were limited to the main channel.

### 2.5 Data assimilation and processing

A single data set was created by combining channel-width measurements at use and available sites. A value of zero was assigned to each available site measurement, and a value of one was assigned to each use site measurement. The river study area associated with each use and available site was also included in order to identify the river system where the measurements were taken. A covariate called “channel break” was created and was assigned a value of one if the maximum unvegetated channel width was <95% of the total channel width and zero if the maximum unvegetated channel width was ≥95% of the total channel width. This covariate was used as an indicator for whether the channel was free of permanently vegetated, mid-channel islands. Please contact corresponding author at baaschd@headwaterscorp.com for more information on data associated with this study.

### 2.6 Relationship between nest incidence and channel width

Logistic regression was used to analyze the relationship between nest incidence (0 = nests absent, 1 = nests present) and channel width metrics. Twelve models including most subsets of main and interaction effects of study area (LPR, Niobrara River and Loup River), total channel width, maximum unvegetated channel width, and channel break were evaluated to determine their usefulness for predicting probability of nesting incidence across the study areas (Table 1). The top model was selected as the most parsimonious model using Akaike's Information Criterion (AIC), with a ΔAIC ≤2.0 (Burnham and Anderson, 2002).

**Table 1:** Akaike Information Criterion (AIC) model selection of an a priori set of models used to evaluate in-channel nesting incidence in the lower Platte, Niobrara, and Loup River study areas, along with AIC model weights. The Null model had an AIC statistic of 1460.93.

Model	AIC	ΔAIC	w
Channel Break × Max Unveg. Width + River System	1363.91	0.00	0.70
Channel Break × Max Unveg. Width	1366.60	2.69	0.18
Channel Break + Max Unveg. Width + River System	1369.00	5.08	0.06
Channel Break × Total Width	1370.68	6.77	0.02
Max Unveg. Width	1370.95	7.04	0.02
Channel Break + Max Unveg. Width	1372.02	8.11	0.01
Channel Break × Total Width + River System	1373.15	9.24	0.01
Channel Break + Total Width	1436.33	72.42	0.00
Channel Break + Total Width + River System	1438.47	74.56	0.00
Total Width	1451.24	87.33	0.00
Channel Break	1460.59	96.68	0.00

### 2.7 Relationship between channel-width metrics

The relationship between total and maximum unvegetated channel width was evaluated using generalized additive models (GAM) assuming a Gaussian (normal) response and a smoothing spline (Hastie and Tibshirani, 1990). GAMs are a type of regression model which allow for nonlinear relationships between the response variable (maximum unvegetated channel width) and a covariate (total channel width). GAMs use a series of polynomials to approximate unknown functional relationships, which made them particularly useful in this case given the theoretical relationship between the variables was unknown. Although GAMs can be used to model nonlinear relationships when the functional form of the relationship is unknown, particular care needs to be taken so the model does not over fit the data. To ensure over fitting did not occur, the target equivalent degrees of freedom for the smoothing spline were varied in integer values from one to five. An additive and interaction effect of river segment (LPR, Niobrara River, Loup River and CPR) were included to test for different relationships between river systems. This resulted in 16 models to fit to the data and the top model was selected as the most parsimonious model with ΔAIC ≤2.0.

Logistic regression was also used to evaluate the relationship between total channel width and channel consolidation (0 = islands absent, 1 = islands present), where consolidation refers to channels free of permanently vegetated islands. A single model that included total channel width and river system as an additive and interaction effect was tested. All analyses were conducted in Program R 3.2.4 (R Core Team, 2016). Predicted relationships were plotted for the best nest-incidence model and channel-width model as well as for the single channel consolidation model.

## 3 Results

### 3.1 Relationship between nest incidence and channel width

A total of 56, 78, and 16 use sites were reported in the LPR, Niobrara River, and Loup River study areas, respectively. Median total channel width at use sites across all river segments was 485 m and median maximum unvegetated channel width was 434 m (Table 2). Ninety percent of use sites occurred in channels with total widths exceeding 352 m and maximum unvegetated channel widths exceeding 265 m. Channel-width measures were generally greater at use sites than available sites.

**Table 2:** Total channel width (m) and maximum unvegetated channel width (m) at systematic available sites and use sites.

Total channel width						
	10th Percentile		Median		90th Percentile	
Study Area	Available	Use	Available	Use	Available	Use
Lower Platte River	347	415	513	536	789	664
Niobrara River	247	390	415	481	602	702
Loup River	136	159	227	264	389	428
All Study areas	200	352	416	485	671	668
**Maximum unvegetated channel width**						
	**10th Percentile**		**Median**		**90th Percentile**	
**Study Area**	**Available**	**Use**	**Available**	**Use**	**Available**	**Use**
Lower Platte River	269	337	403	496	548	608
Niobrara River	158	318	323	431	473	549
Loup River	120	136	201	181	318	329
All Study areas	156	265	325	434	495	588

**Fig. 4:**
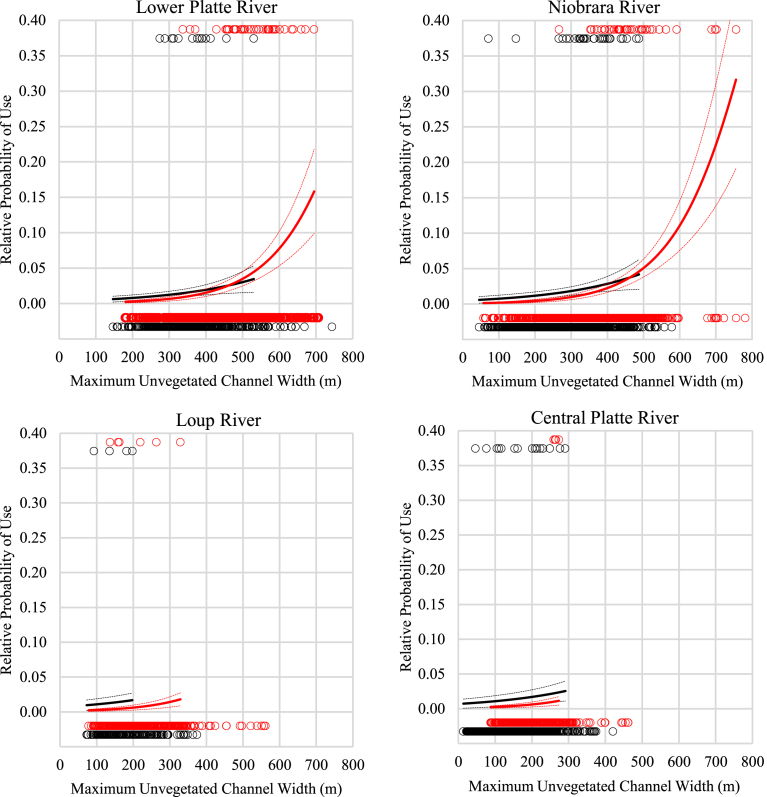
Predicted probability of use for consolidated (red) and unconsolidated channels (black) compared to maximum unvegetated channel width for the lower Platte (upper left), Niobrara (upper right), and Loup (lower left) segments. Predicted probability of nesting incidence is only plotted to the maximum unvegetated channel widths observed at nesting locations within each river system. Open circle points show maximum unvegetated channel widths for use sites (Red) and available (Black) sites. Predicted probability of use was plotted for the central Platte segment area (lower right) for comparison, however, data from the central Platte were not used to estimate model parameters.

The logistic regression models with the highest predictive ability contained the effects of maximum unvegetated channel width and an interaction between maximum unvegetated channel width and channel break (Table 1). We found probability of nesting increased as maximum unvegetated channel width increased and rapidly increased once maximum unvegetated channel width reached approximately 500 m for consolidated channels (Fig. 4). In unconsolidated channels, the probability of nesting also increased with maximum unvegetated channel width. However, maximum unvegetated widths in unconsolidated channels were generally much lower than 500 m. The lower Platte and Niobrara Rivers had the highest likelihood of use of any study area with probability of use maximized when maximum unvegetated channel width was approximately 700 m, while the Loup River had very low likelihood of use. Utilizing the predictive model, we found the CPR study area was predicted to have a very low probability of nesting, similar to nesting likelihood on the Loup River, principally due to a lack of maximum unvegetated channel widths greater than 400 m.

### 3.2 Relationship between channel-width metrics

**Fig. 5:**
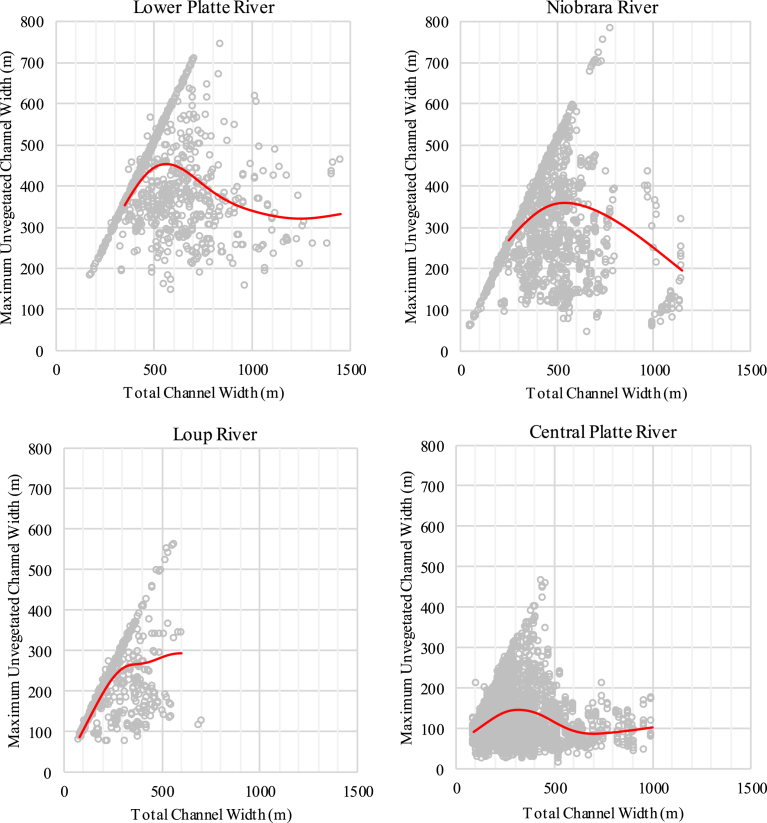
Relationship between total channel width and maximum unvegetated channel width for the lower Platte (upper left), Niobrara (upper right), Loup (lower left), and central Platte (lower right) River study segments.

The function response between total channel width and maximum unvegetated channel width was similar across study areas (Fig. 5). Our model predicted maximum unvegetated channel width would increase until total channel width exceeded approximately 500 m in all river systems besides the CPR, where maximum unvegetated channel width increased until total channel width exceeded approximately 300 m (Table 3; Fig. 5). In channels wider than 500 m, or wider than 300 m in the CPR, maximum unvegetated channel width decreased in spite of increasing total channel width due to the increasing occurrence of vegetated islands in wider channels. However, the underlying data was highly variable as segments of the LPR channels as narrow as 335 m contained vegetated islands and channels as wide as 700 meters were found to be fully consolidated (Fig. 5). The width relationship and underlying data in the Niobrara study area was very similar to the lower Platte River. The general relationships for the Loup and CPR study areas were also similar, but overall widths were narrower with few consolidated channels occurring when total channel width exceeded 350 m.

**Table 3:** Akaike Information Criterion (AIC) ranked models for analysis of the relationship between total channel width and maximum unvegetated channel width in the lower Platte River, Niobrara River, Loup River and central Platte River study areas. The Null model had an AIC statistic of 125,465.76.

Model	AIC	ΔAIC	w
Total Width (df = 5) × River System	118,598.42	0.00	1.00
Total Width (df = 4) × River System	118,649.13	50.71	0.00
Total Width (df = 3) × River System	118,764.62	166.19	0.00
Total Width (df = 2) × River System	119,273.97	675.55	0.00
Total Width (df = 1) × River System	120,701.67	2103.24	0.00
Total Width (df = 5) + River System	119,404.96	806.53	0.00
Total Width (df = 4) + River System	119,447.14	848.71	0.00
Total Width (df = 3) + River System	119,523.50	925.07	0.00
Total Width (df = 2) + River System	119,814.74	1216.31	0.00
Total Width (df = 1) + River System	121,072.19	2473.76	0.00
Total Width (df = 5)	125,542.48	6944.05	0.00
Total Width (df = 4)	125,589.33	6990.91	0.00
Total Width (df = 3)	125,695.64	7097.21	0.00
Total Width (df = 2)	126,053.12	7454.70	0.00
Total Width (df = 1)	127,709.90	9111.48	0.00

**Fig. 6:**
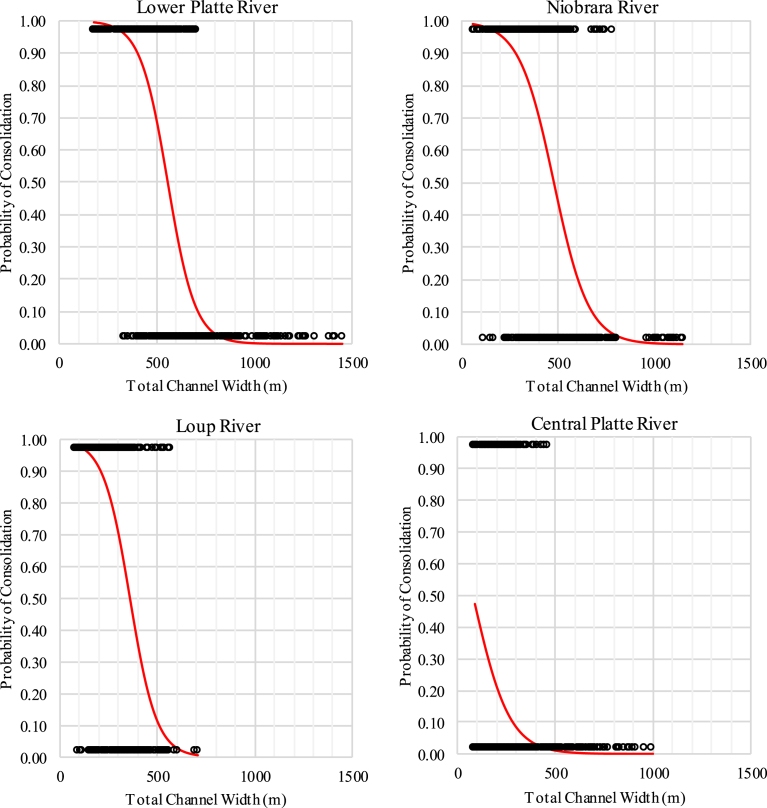
Relationship between total channel width and probability that channel will be free of vegetated islands (consolidated) for the lower Platte (upper left), Niobrara (upper right), Loup (lower left), and central Platte (lower right) River study segments.

The relationship between total channel width and probability of channel consolidation (i.e., free of vegetated islands) indicated a decreasing probability of consolidation with increasing total channel width (Fig. 6). We estimated there is a 50% probability of consolidation when total channel width exceeded 560 m in the LPR study area, 480 m in the Niobrara River study area, 360 m in the Loup River study area, and less than 100 m in the CPR study area.

## 4 Discussion

From a species habitat perspective, our findings were consistent with both Ziewitz et al. (1992) and Jorgensen et al. (2012) as we found probability of nesting incidence increased with increasing maximum unvegetated channel width for all segments with the probability of use increasing rapidly once width reached approximately 500 m (Fig. 4). From a geomorphic perspective, our findings were also consistent with Elliott's (2011) geomorphic analysis as we found a decreasing probability of the channel being free of vegetated islands with increasing total channel width for all segments (Fig. 6). The varying channel widths and width-consolidation relationships by segment were expected given differences in hydrology and the controlling influence of flow on alluvial channel form (Richards, 1982; Schumm, 2007; Bridge, 2009).

Integrating species- and geomorphic-centric perspectives provided additional insights that could be used to predict species' response to actions that affect channel widths in these segments. First, segments like the central Platte River and Loup River, where total channel widths are generally below 300 m, have a low probability of species use, regardless of the presence of sandbar habitat. In the wider lower Platte River and Niobrara River segments, the widest channels free of permanently vegetated islands appear to be extremely important from a species use perspective. This reflects the tradeoff between increasing probability of use and decreasing probability of consolidation (absence of permanently vegetated islands) with increasing total channel width. Consolidated channels with total widths of 500–800 m are relatively rare, but have the highest probability of use. Therefore, actions reducing channel width in these locations would likely have the greatest negative impact on least tern and piping plover use. Conversely, actions in the widest channels (total width) of these segments would likely have little impact on use as these channels cannot be maintained free of vegetated islands through natural processes.

If management actions are contemplated to increase species use in the lower Platte River or Niobrara River segments, removal of permanently vegetated islands in areas where total channel width ranges from 500–800 m wide may greatly improve probability of use. However, as total width increases, there would be a corresponding tradeoff between increasing probability of use and decreasing probability the channel will remain free of vegetated islands. Further exploration of differences in physical characteristics of consolidated and unconsolidated channels of similar widths would be beneficial.

As demonstrated above, integration of species-centric and geomorphic-centric analyses provides additional information for decision making. In this case, helping to focus impact assessments and management efforts towards channel configurations that are wide enough for a high probability of species use and yet narrow enough to be maintained free of permanently vegetated islands by natural riverine processes. It also highlights the care that must be taken when interpreting the results of investigations that utilize common physical habitat terms such as channel width. Channel configurations utilized by least terns and piping plovers may be simultaneously identified as wide from species habitat perspective and narrow from a geomorphic perspective. Cross-disciplinary investigations like the one presented here offer one way to reduce the potential for miscommunication between ecological and physical science practitioners.

## Declarations

### Author contribution statement

Jason M. Farnsworth, David M. Baasch, Patrick D. Farrell: Conceived and designed the experiments; Performed the experiments; Analyzed and interpreted the data; Contributed reagents, materials, analysis tools or data; Wrote the paper.

### Funding statement

This work was supported by the Platte River Recovery Implementation Program.

### Competing interest statement

The authors declare no conflict of interest.

### Additional information

No additional information is available for this paper.
